# Application of Strain Selection Technology in Alcoholic Beverages: A Review

**DOI:** 10.3390/foods13091396

**Published:** 2024-05-01

**Authors:** Xiaodie Chen, Chuan Song, Jian Zhao, Zhuang Xiong, Lianxin Peng, Liang Zou, Caihong Shen, Qiang Li

**Affiliations:** 1 Key Laboratory of Coarse Cereal Processing, Ministry of Agriculture and Rural Affairs, Sichuan Engineering & Technology Research Center of Coarse Cereal Industrialization, School of Food and Biological Engineering, Chengdu University, Chengdu 610106, China; cxd0512@126.com (X.C.); xiongzhuang2000@126.com (Z.X.); penglianxin@cdu.edu.cn (L.P.); zouliang@cdu.edu.cn (L.Z.); 2 Luzhou Laojiao Co., Ltd., Luzhou 646000, China; songchuan@lzlj.com; 3 National Engineering Research Center of Solid-State Brewing, Luzhou 646000, China; 4 Postdoctoral Research Station of Luzhou Laojiao Company, Luzhou 646000, China; 5 School of Life Sciences, Sichuan University, Chengdu 610041, China; zj804@163.com

**Keywords:** strain selection technology, alcohol beverage, microbial diversity, food processing, *Saccharomyces cerevisiae*

## Abstract

The diversity of alcohol beverage microorganisms is of great significance for improving the brewing process and the quality of alcohol beverage products. During the process of making alcoholic beverages, a group of microorganisms, represented by yeast and lactic acid bacteria, conducts fermentation. These microorganisms have complex synergistic or competitive relationships, and the participation of different microorganisms has a major impact on the fermentation process and the flavor and aroma of the product. Strain selection is one of the key steps. Utilizing scientific breeding technology, the relationship between strains can be managed, the composition of the alcoholic beverage microbial community can be improved, and the quality and flavor of the alcoholic beverage products can be increased. Currently, research on the microbial diversity of alcohol beverages has received extensive attention. However, the selection technology for dominant bacteria in alcohol beverages has not yet been systematically summarized. To breed better-quality alcohol beverage strains and improve the quality and characteristics of wine, this paper introduces the microbial diversity characteristics of the world’s three major brewing alcohols: beer, wine, and yellow wine, as well as the breeding technologies of related strains. The application of culture selection technology in the study of microbial diversity of brewed wine was reviewed and analyzed. The strain selection technology and alcohol beverage process should be combined to explore the potential application of a diverse array of alcohol beverage strains, thereby boosting the quality and flavor of the alcohol beverage and driving the sustainable development of the alcoholic beverage industry.

## 1. Introduction

The production of beverages through alcoholic fermentation dates back thousands of years [1,2]. Microorganisms ferment substances containing starchy and sugary raw materials to produce an alcoholic component, which creates alcoholic beverages. Yellow wine, wine, and beer are the world’s three major alcoholic beverages [3,4]. Of these, beer dominates the global alcoholic beverage market [5]. Consumed in regions such as the Americas, Asia, and Europe, with the highest per capita consumption in the Czech Republic at 6.77 L, the beverage generated revenues of USD 563.9 billion in 2022 [6].

Different types of alcoholic beverages require specific microbial species to participate in the fermentation process. These microorganisms mainly include yeasts, bacteria, and molds [7]. The brewing of wine mainly depends on yeast, which transforms the sugars in grapes into alcohol and carbon dioxide. Different types of yeast can affect the flavor and mouthfeel of wine; for example, some yeasts can produce special aromas and tastes, such as oaky and fruity aromas [8]. In addition, some bacteria, such as lactic acid bacteria and acetic acid bacteria, also participate in the wine fermentation process [9]. In the beer brewing process, in addition to yeast, *Lactobacilli*, *Bifidobacteria*, and *Enterococci* also participate [10]. These microorganisms can produce substances such as acetic acid and lactic acid, thereby affecting the taste and texture of the beer. Therefore, it is very important to study and breed microbial strains suitable for the brewing of different types of wines. Strain breeding technology is the use of various biotechnologies to modify the genetic material of microorganisms to avoid or alleviate the adverse genetic traits of these microorganisms. Through screening and cultivation of microbial strains in the alcoholic beverage process, strains with excellent traits, such as high alcohol tolerance, low acidity, and high enzyme activity, can be obtained [11,12,13,14]. These strains can effectively improve the taste, aroma, and color of alcoholic beverages, as well as their quality and flavor.

At present, most studies have focused on elucidating the effects of the raw materials, fermentation processes, etc. used in wine making on the diversity of microorganisms in alcoholic beverages. Research on breeding technologies for relevant strains is also limited, and there are few studies on the application of selective breeding technology to the diversity of microorganisms in alcoholic beverages. Furthermore, there is a lack of a systematic summary on this topic. Therefore, this paper reviewed the microbial diversity characteristics and breeding technologies of related microbial species in the world’s three major brewing alcohols: beer, wine, and yellow wine. It also analyzed the application of strain selection technology in the study of microbial diversity in alcoholic beverages, with the aim of providing a new theoretical basis for the study of microbial interrelationships and regulatory mechanisms in the fermentation process.

## 2. Type and Diversity Characteristics of Microorganisms in Alcoholic Beverages

### 2.1. Types and Diversity Characteristics of Microorganisms in Yellow Wine

Yellow wine is made from grains used as raw materials, followed by steaming, adding jiuqu, saccharification, and fermentation, and finally, pressing and filtering [15]. A variety of microorganisms involved in the fermentation process of yellow wine produce hundreds of metabolites that contribute to its distinctive aroma. The microorganisms present in yellow wine greatly determine the taste and excellence of the end product. Due to its remarkable flavor, low alcoholic content, and abundance of nutrients like peptides, amino acids, oligosaccharides, and vitamins, yellow wine is widely consumed in Asia. At the same time, the phenolic compounds and mineral components in yellow wine have numerous health benefits, including decreasing cholesterol levels, antioxidant activity, and slowing down the aging process [16,17,18], and are also good for preventing cardiovascular disease and cancer [19,20].

The yellow wine brewing process is a metabolic activity of microorganisms, and yeast, molds, and bacteria are the three most common types of microorganisms. The molds in yellow wine are mainly sieved from the jiuqu, and different molds are one of the causes of the unique local flavors of yellow wines [21]. Some studies have shown that *Rhizopus* and *Aspergillus* play a key role in yellow wine. They can both produce various enzymes. *Rhizopus* can produce high-activity amylases and glucoamylases, and these enzymes have a great impact on the saccharification of the yellow wine [22]. In addition, some *Rhizopus* species can process alcohol under specific conditions to produce flavor compounds such as 2-phenylethanol, ethyl hexanoate, and ethyl lactate [23]. *Aspergillus* can produce acid protease and carboxypeptidase, which can break down proteins in rice into peptides and amino acids, thus providing a source of nitrogen that can be utilized by yeast [24]. These substances can be used to provide nutrients for the growth of yeast and to synthesize flavor compounds [25]. In general, *Aspergillus* is a common filamentous fungus, and it is present in all stages of the fermentation process of Maiko Yellow Wine [26]. The utilization of molds as a starter can effectively enhance the production stability, amino acid content, alcohol content, and volatile flavor substances of yellow wine, and promote the industrialization of yellow wine [27]. Liu Y et al. [28] found that the use of a mix of fungi (*Aspergillus niger* A20, *Mucor pusillus* M05, *Rhizopus chinensis* R01, and *Saccharomyces cerevisiae* S10) as a starter, with the addition of α-amylase (AM), glucoamylase (GAM), and acid protease (AP), could significantly increase the alcohol content and enrich the flavor components of yellow wine.

Yeast is also the main microorganism in the production of yellow wine, and its quantity and quality directly affect the flavor and quality of the yellow wine [29]. The winemaking process is not a “single species” fermentation process [30]. *S. cerevisiae* (cultured or naturally occurring) has a great advantage in the fermentation process. However, the number of non-Saccharomyces yeasts in yellow wine is often greater than that of *Saccharomyces cerevisiae*, and their adaptability to special environments and active growth state give them an advantage in competition. In the studies of Pretorius et al. [31,32,33], they found that the screened non-Saccharomyces could efficiently utilize sugars, increase the yield of desired volatile esters, promote the release of grape terpenes, and produce glycerol, thereby improving the flavor and other sensory properties of the wine. An excellent yellow wine yeast should have strong fermentation power and produce essential and coordinated flavor compounds. Huang et al. [34] reported that the most common non-yeast fungi in yellow wine were *Geotrichum*, *Issatchenkia*, *Rhodotorula*, *Debaryomyces*, *Wickerhamomyces*, *Candida*, *Clavispora*, and *Blastobotrys*. Sequential fermentation using both *Saccharomyces cerevisiae* and non-*Saccharomyces* can produce a Chardonnay wine with a lower alcohol content and good chemical volatilities [35].

Compared to fungi, such as yeast and mold, the role of bacteria in yellow wine has yet to be studied. The number of various types of bacteria in yellow wine was far higher than in that of yeast and mold. Ping et al. [36] used Illumina MiSeq pyrosequencing to monitor bacterial changes during the yellow wine brewing process, and the kinetics of volatile compounds were monitored in combination with HS-SPME/GC-MS to assess the effect of the bacteria on the formation of volatile compounds. The results showed that *Thermoactinomyces*, *Pseudomonas* spp., *Monascus*, *Lactococcus*, and *Bacillus* played key roles in the synthesis of various volatile compounds in Shaoxing Maiqu Yellow Wine. Through traditional PCR-DGGE and high-throughput sequencing (HTS) analysis, it was found that the most dominant bacterial genera in the yellow wine brewing process were *Bacillus* and *Lactobacillus* [34,37,38]. As brewing progressed, the floral structure changed significantly, with the most significant abundance of *Bacillus* and *Lactobacilli*. During the fermentation process, *Bacillus* can produce a large amount of hydrolytic enzymes, which can form aromatic substances such as diacetyl and nitrogen-containing compounds [39]. In addition, Bacillus is a type of microorganism that can survive in adversity in its spore form and can perform secondary fermentation under high concentrations of ethanol to produce flavor compounds. LAB can produce a variety of antibacterial substances that can effectively inhibit pathogens and toxin-producing microorganisms. Organic acids prepared from lactic acid can be used as precursors for the formation of flavor compounds. Research has demonstrated that, in the wine-making process, the content of LAB is positively proportional to the content of organic acids [40].

**Figure 1:**
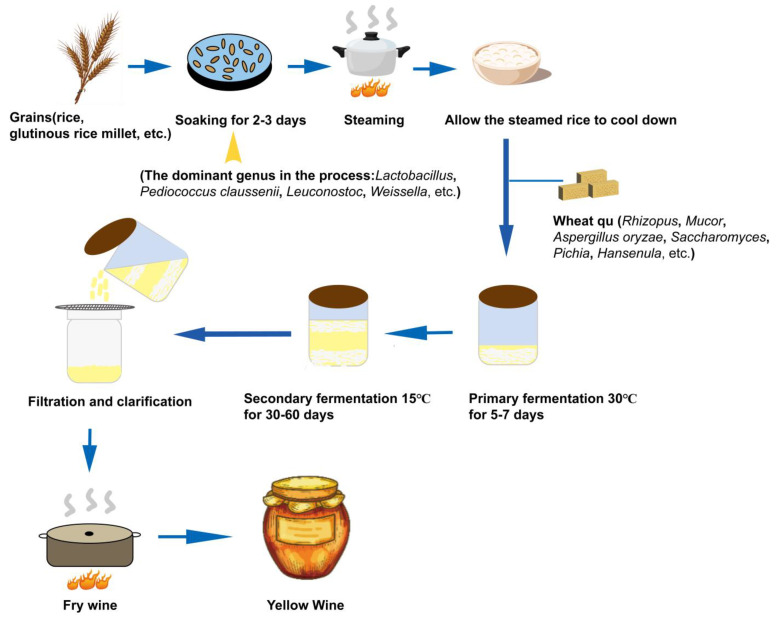
Process flow of yellow wine brewing.

In the brewing process of yellow wine, the main ingredients are actually a mixture of molds, yeasts, and bacteria after fermentation. Generally, molds perform saccharification, lactic acid bacteria metabolize acidic substances, and yeasts ferment and metabolize alcohol. Process enhancements and the interaction between yeast and jiuqu during fermentation have complicated yellow wine production, making it a complex biological reaction system. The complex microbial communities in yellow wine not only produce various enzymes and degrade macromolecules, but also promote the metabolism and synthesis of flavor substances in yellow wine, including organic acids, free amino acids, and esters, endowing it with rich nutrients and unique flavors [41,42,43]. As fermentation progresses and the environment changes, the dominant microorganisms and strains continue. During this period, fungi and bacteria secrete various enzymes, such as proteases, amylases, and esterases, to hydrolyze proteins and starches, and yeast converts sugars into ethanol. Acetic acid bacteria (AAB) and lactic acid bacteria (LAB) produce organic acids to lower the pH [44]. Meanwhile, nitrogenous toxins produced by microbial metabolism, such as biogenic amines (BAs) and ethyl carbamate (EC), are often detected during yellow wine fermentations [45,46]. EC is very harmful; molecular biology studies have proven it to be mutagenic and carcinogenic in mice, rats, and monkeys [47,48]. During the industrial fermentation process, *S. cerevisiae* produces EC, in addition to ethanol and flavoring substances. In yellow wine fermentation, urea and ethanol, both of which can be produced by *S. cerevisiae*, are the precursors of EC [49]. Yeast produces urea from arginine. Urea can then react with ethanol to form urethane. Bacteria or yeast can produce BA through the process of decarboxylation of amino acids, which can occur during fermentation or aging. Bacteria and yeast decarboxylate amino acids, such as tyrosine, histidine, and arginine, to form biogenic amines, such as histamine, tyramine, and putrescine. In yellow wine, histamine is the most toxic, and it is synthesized by *Staphylococcus albus* and *Lactobacillus hilgardii* through histidine decarboxylation [50]. Putrescine biosynthesis by *Enterococcus faecalis*, *Enterococcus sclerosus*, and *Enterococcus faecium* occurs through the agmatine deamination pathway [51]. *Lactococcus* found in dairy products is able to manufacture putrescine; however, glucose and lactose have the ability to suppress this production [52]. Production of tyramine by *Lactobacillus brevis* IOEB 9809 occurs through decarboxylation of tyrosine [22]. The types of bacteria present in wine also affect the production of biogenic amines. Luo et al. [53] used the gene prediction function method to study the effects of different bacterial species on the production of an amino acid decarboxylase in the fermentation of yellow wines. The most abundant species were *Citrobacter*, *Acinetobacter*, *Lactobacillus*, *Bacillus*, *Pseudomonas*, and *Enterobacter*. Therefore, in wine production, safe fermentation strains should be selected to minimize the formation of harmful substances in the wine and ensure the quality and safety of the product. Figure 1 shows the brewing process of a yellow wine, showing the dominant microorganisms present in different stages of brewing.

### 2.2. Types and Diversity Characteristics of Microorganisms in Wine

Wine is an alcoholic beverage made from fermented grapes with an alcohol content ranging from 8 to 15%. There are many types of wine produced with varying varieties, planting regions, planting techniques, and fermentation environments. Moderate drinking of wine is good for the human body. For example, wine is rich in polyphenols, which can be beneficial for health, including the prevention of cancer effects and cardiovascular diseases [54]. Wine production is inextricably linked to microorganisms. The production of wine from grape juice is the consequence of the combination of yeasts, bacteria, and filamentous fungi [55]. Even if bacteria are usually fewer than yeasts, they can still have a major influence on the taste and aroma of the wine. The bacteria associated with wine making are mainly LAB, AAB, and Bacillus [56,57,58,59]. For example, LAB can convert malic acid to lactic acid, which can make wine taste smoother and have a lower acidity. The lactic acid bacteria mainly include *Lactobacillus*, *Oenococcus*, *Pediococcus,* and *Leuconostoc* [60], and *Oenococcus oeni* (*O. oeni*) among the bacteria participating in malolactic fermentation (MLF), and MLF is the main biological acid reduction method in wine production [61]. Under the action of *O. oeni*, malic acid is converted to lactic acid, with carbon dioxide being released in the process. Most lactic acid bacteria (including *O. oeni*) can produce glucosidase, and an adequate amount of glucosidase can promote the development of varietal aromas in wine [62,63,64]. In addition to glucosidases, many studies have used immobilized glycosidases to enhance wine aroma [65,66,67]. For example, β-glucosidase, α-arabinosidase, and α-rhamnosidase were immobilized on acrylic beads to promote the release of wine aromas [68]. On the other hand, for wine, most bacteria can have a negative effect on the quality of the wine. For example, AAB can produce acetic acid. If there is a high concentration of acetic acid, it will impart a sour taste and spoil the wine. The acetic acid bacteria that have been found to be harmful to wine in wine making include *Acetobacter aceti* [69], *Acetobacter pasteurianus* [70], and *Gluconobacter oxydans* [71]. They can oxidize ethanol to acetic acid, resulting in an increase in the volatile acids in the wine [72]. In the wine-making process, because microbial or environmental factors can cause wine to spoil, wine makers will continue to use SO_2_ to reduce spoilage, but this may cause some health problems and changes in the wine’s sensory characteristics. Izquierdo et al. [73] explored the ability of the KAgC complex to control the production of AAB in spoiled wines. The study showed that, after wine supplemented with KAgC complex had been incubated for 72 h, the amount of AAB in the wine was negligible. The elimination of AAB in wine reduces the use of SO_2_ and ensures the quality of the wine. The harm of Bacillus to wine consists of mainly making the wine turbid. Bae et al. [74] isolated Bacillus from a grape juice and inoculated it into wines and grape juices. The study found that Bacillus was still alive, but did not grow.

The process of wine fermentation is largely attributed to the yeast microorganisms. The sugars of the grapes are consumed by them to make alcohol and carbon dioxide. In the process, alcohol, aldehydes, and ester metabolites are generated. Different yeast strains can produce different tastes and aromas in wine, and wine makers often select specific strains to achieve desirable characteristics. At present, there are approximately 150 species of yeast generally involved in the wine fermentation process, including 25 genera, among which are the genera *Saccharomyces*, *Candida*, *Pichia*, *Hanseniaspora*, *Schizosaccharomyces*, *Dekkera*, *Metschnikowia*, and *Zygosaccharomyces*, including ten genera of yeasts [75,76]. *Saccharomyces* sp. is an essential microorganism in the grape fermentation process. Yeast not only inhibits the growth of mold on the surface of grapes, but also produces more than 400 volatile aroma substances during fermentation [8]. In the wine-making industry, yeasts are divided into two categories: *S. cerevisiae* and non-*S. cerevisiae*. Oliveira et al. [77] studied the fermentation ability of *S. cerevisiae* and non-*S. cerevisiae*, and the effect on the wine’s flavor was studied. GC-MS analysis showed that non-*S. cerevisiae* produced more types of volatile substances, had higher concentrations, and had a more prominent aroma than that of *S. cerevisiae*. The wine production rates between the two were similar.

Yeasts other than *S. cerevisiae* also have some disadvantages, such as low alcohol production. Therefore, the use of mixed fermentation to enable different strains to “draw from each other’s strengths” is a current research hot spot. *Pichia kluyveri*, one of the most concerning strains of non-*S. cerevisiae*, can effectively improve the floral and fruity aromas in wine [78]. However, *P. kluyveri* has a poor alcohol tolerance and cannot complete alcoholic fermentation on its own. It is usually mixed with *S. cerevisiae* to improve the quality of the wine. Dutraive et al. [79] showed that, compared to wines purely cultured by *S. cerevisiae*, the concentrations of total esters, glycerol, and valeric acid in the cofermentation of P. *kluyveri* with *S. cerevisiae* were higher; Ge et al. [80] found that *P. kluyveri* could significantly increase the contents of linalool, p-xylene, and nerol oxide in wine.

**Figure 2:**
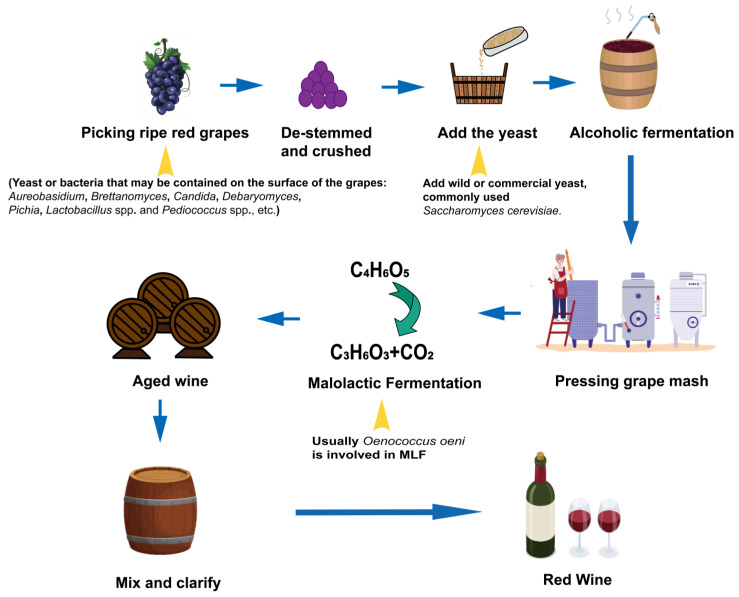
Wine-making process.

Fungi are also present in wine, although they are usually of less importance than bacteria and yeasts. Mold is a filamentous fungus that can contaminate grapes in a humid environment, reducing yields and affecting quality. In addition, aging oak barrels, both interior and exterior, and the corks of wine bottles are prone to mold infections. If fungi are present in high concentrations, they can cause spoilage and off flavors in the wine. However, mold is not all bad for wine. Noble rot, which originated in Hungary, is produced by *Botrytis cinerea*, known as “noble mold”. Research has demonstrated that the quality of Amarone della Valpolicella wines can be subject to change depending on the occurrence of ideal climate conditions for mold infestation. The noble rot can significantly alter the aroma composition (alcohols, aldehydes, fatty acids, and lactones) in wine [81,82]. Although there are more studies on molds used in noble rot winemaking, molds still have great research potential in the rest of the wine-making industry. Figure 2 shows the wine-making process, illustrating the dominant microorganisms that exist at different stages of wine making.

### 2.3. Types and Diversity Characteristics of Microorganisms in Beer

Beer is a popular alcoholic beverage, consumed worldwide. It is a combination of water, malted barley, hops, and yeast [83]. Yeast plays a critical role in beer production by converting the sugars in germinated barley into alcohol and carbon dioxide. The type of yeast used in beer production can greatly affect the taste and aroma of the final product. In addition to yeast, other microorganisms, such as bacteria and fungi, may also be present in beer and affect its quality and safety.

**Figure 3:**
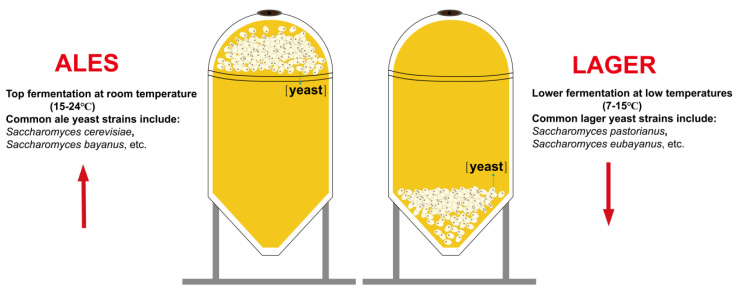
Schematic diagrams of top-fermenting and bottom-fermenting craft beers. The points of the red arrows in the diagram correspond to the positions of the yeast in top-fermented and bottom-fermented beers, respectively.

Beer production utilizes two primary types of yeast: ale yeast and lager yeast [84]. Ale yeast is a top-fermenting yeast that ferments at temperatures ranging from 15 to 24 °C [85]. It produces fruity and spicy flavors and is used in the production of ales, stouts, and porters. Some common ale yeast strains include *Saccharomyces cerevisiae* and *Saccharomyces bayanus* [86]. Lager yeast is a bottom-fermenting yeast that ferments at temperatures between 7 °C and 15 °C [85]. It produces a clean and crisp flavor and is used in the production of lagers and pilsners. Some common lager yeast strains include *Saccharomyces pastorianus* and *Saccharomyces eubayanus*. *Saccharomyces pastorianus* are hybrids of *S. cerevisiae* and *S. eubayanus* [87]. Figure 3 shows schematic diagrams of two types of craft beer: top-fermented and bottom-fermented.

Bacteria can also be present in beer, but they are usually undesirable and can cause off-flavors and spoilage. The most common bacteria in beer are lactic acid bacteria and acetic acid bacteria. Lactic acid bacteria (LAB) are responsible for the production of lactic acid, which gives beer its sour taste. For example, some spoilage LAB strains found in beer include *Lactobacillus brucei*, *Lactobacillus plantarum*, and *Pediococcus damnosus* [88]. Acetic acid bacteria (AAB) are responsible for the production of acetic acid, which can give beer its vinegary flavor. AAB contamination is usually the result of inadequate hygienic conditions during the brewing or aging process. Some common AAB strains found in beer include *Acetobacter aceti* and *Gluconobacter oxydans* [89].

The presence of fungi in beer can also cause spoilage. The most common fungi in beer are wild yeasts and molds. Wild yeast is a non-sugar yeast that can produce a strange and sour taste. Mold can also be present in beer, usually as the result of contamination by grains or hops. Mold contamination can cause odors and spoilage. Some of the common molds found in beer include Aspergillus and Penicillium [90].

The diversity of microorganisms in beer can greatly affect its flavor, aroma, and quality. In recent years, the diversity of yeast strains used in beer production has increased, resulting in a wider range of beer styles and tastes. In addition to traditional beers and lagers, brewers are now using various non-sugar yeasts and mixed cultures to produce unique and complex flavors. Mixed-culture fermentation involves the use of different combinations of yeasts and bacteria to ferment beer, and is usually used for the production of sour beers. Our study showed that the presence of *Lactobacillus brevis* BSO464 and *Lactobacillus plantarum* had a positive effect on the composition of flavor-active substances in sour beer [91]. In addition, fermentation with *S. cerevisiae* could control the production of a sour beer and shorten the production time. This beer has grown in popularity in recent years. In addition to yeast and bacteria, the use of hops in beer production also affects the diversity of microorganisms present in beer. Hops have antibacterial properties that can help inhibit the growth of unwanted bacteria and fungi. However, they also affect the growth and activity of yeasts, resulting in changes in the taste and aroma of the beer. Table 1 lists the dominant microorganisms in some typical wine varieties for brewing.

## 3. Strain Selection Techniques

Microbial strains are the key to determining the industrial value of fermentation products and the success of fermentation projects. Therefore, strain selection is very important for wine making. The use of excellent yeast strains can control the amount of byproducts formed and improve the flavor and quality of the wine. In the production of beer, the screening method is usually used to select yeast strains that can control the production amount of byproducts, especially the content of diacetyl and higher alcohols, which have a great impact on the quality of beer [113]. Vion et al. [114] used marker-assisted selection (MAS) to enhance malic acid consumption by *S. cerevisiae* in grape must, and MAS proved to be effective in selecting industrial *S. cerevisiae* with unusual characteristics. Bellon et al. [115] chose to utilize interspecies hybridization for wine yeast breeding by adding non-*S. cerevisiae* genomes to commercial wine yeasts, which can generate new phenotypes and develop new wine styles.

At present, the breeding technologies for wine are mainly focused on two directions. One is to use natural microorganisms to differentiate strains suitable for different wines. Researchers generally look for stressed, adaptable microorganisms that can tolerate unfavorable conditions, such as low temperatures and a low pH. These microorganisms not only reflect the quality characteristics of wine, such as color, taste, and aroma, but also have the operability of large-scale production and stable quality. Second, modern bioengineering technology is used to screen high-yielding strains to improve the fermentation effects. The modifications are mainly performed on yeast. However, any genetic modification of yeast is strictly regulated under the gene technology laws of many countries. These laws stipulate that genetically modified yeast strains must meet specific requirements to ensure their safety and efficacy before they can be approved for industrial use. It has been reported that almost all yeast used in the brewing industry is derived from the two most basic strains of yeast in the laboratory: *Saccharomyces cerevisiae* and *Saccharomyces bayanus* (cold winestain yeast). To reduce urethane in alcoholic beverages, scientists successfully gene-edited yeast using CRISPR/Cas9 technology [116].

Since genetically engineered microorganisms may be more sensitive to certain environmental conditions, thus affecting their ecological adaptability in industrial production, a strict safety assessment is needed, so the breeding of beer and yellow wine strains is often based on traditional breeding techniques, such as physical mutagenesis, chemical mutagenesis, and protoplast fusion techniques. The protoplasts of *S. cerevisiae* were treated with a compound of mutagenesis, and the screened mutant strains could greatly increase the production of metabolites.

### 3.1. Natural Selection

**Figure 4:**
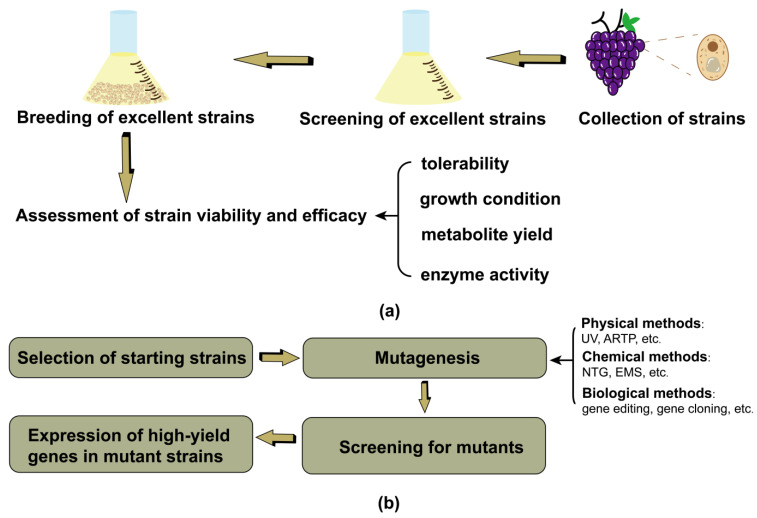
(**a**) General steps of natural selection; (**b**) the basic process of induced mutation. The arrows in the diagram indicate the sequence of the strain selection process.

Strains with excellent characteristics are obtained through natural mutation at multiple levels, including tissues, cells, protoplasts, and microorganisms, and these traits are fixed in the genes. Natural breeding is the selection of organisms during the reproductive process under environmental conditions. The removal of mutations that are unfavorable for their survival and development, and the retention of only the beneficial mutations for semi-conservative DNA replication of the organisms themselves, are retained. It is important to clarify that mutation events occurring during growth do not constitute “breeding”, but are separate phenomena that occur naturally or are induced in the laboratory. The correction and photorepair of the corrective enzyme system, excision repair, and mechanisms such as recombination repair and inducible repair can both cause mutations. Pérez-Coello et al. [117] isolated a total of 392 yeast strains from three vineyards in the La Mancha region of Spain. The yeast strains 27, 41, and 230 were found to be able to synthesize high levels of hexyl acetate and amyl acetate, which significantly enhanced the fruity aroma of wines. They also produced high concentrations of cis-3-hexen-1-ol and 1-hexanol, which enhanced the herbal aroma of wines. Zheng et al. [118] selected R-SYB082, which has acidic urea degradation characteristics, from fermented food. This strain can be used to isolate a urea acetate amidohydrolase (UAH), an enzyme that degrades urea and EC, with the removal rate of EC from Chinese yellow wine reaching 90.7%. The basic steps of natural selection are shown in Figure 4a.

### 3.2. Induced Mutation

Induced mutation is the selection of an elite variety that meets the needed traits through artificial mutagenesis and physical and chemical means. The basic process of induced mutation is shown in Figure 4b. The most commonly used physical mutagenesis technique is UV mutagenesis; the UV spectrum is the same as the absorption spectrum of nucleic acids in cells, with the absorption of DNA at 250 nm being the maximum [119]. After the purines and pyrimidines of the DNA and RNA of the strain absorb UV light, the DNA molecule forms a pyrimidine dimer; that is, two adjacent pyrimidines are covalently linked and dimerized, resulting in deformation of the double-stranded structure and hindering the normal pairing between bases [120], which can cause mutations or death. In addition, pyrimidine dimer formation hinders the double-stranded unwinding, thereby affecting DNA replication and transcription [121]. In addition, there are microwave, laser, and plasma mutagenesis techniques. The use of some chemical mutagens mainly includes some chemical drugs, such as certain alkylating agents, base analogs, and antibiotics. These compounds directly react with molecules such as pyrimidine, purine, and phosphoric acid in nucleotides, causing mutations in the genes of microorganisms. Researchers Yi et al. [122] used the synergistic mutagenesis method of ultraviolet (UV) and diethyl sulfate (DES) to identify a second-generation bio-*Saccharomyces cerevisiae* with high ethanol yield, high ethanol tolerance, temperature tolerance, and glucose tolerance. Yang et al. [123] used the same mutagenesis method to obtain the mutant YN81 from the parent strain CS31 used in high-specific-gravity craft beer brewing. The mutant YN81 had a high ethanol tolerance and a higher fermentation ability and alcohol production capacity.

### 3.3. Crossing Techniques

**Figure 5:**
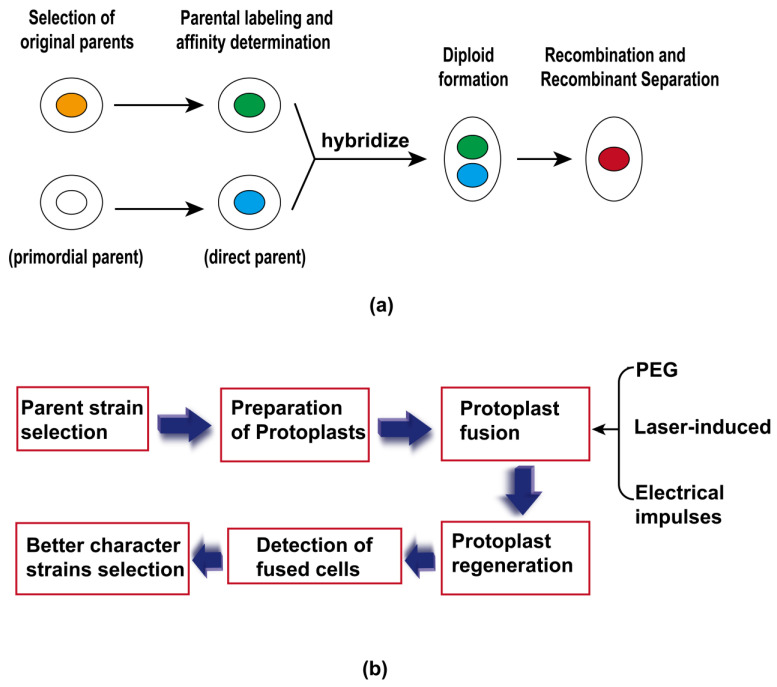
(**a**) General steps of microbial hybridization breeding; (**b**) the basic process of protoplast fusion.

The crossing technique refers to the rapid acquisition of new excellent strains through the crossing and mating of two strains with different genotypes and the use of the principles of genetic variation and gene recombination. However, it is important to note that in practice, challenges often arise, as diploid strains typically need to be converted into haploids before successful crossings can occur. While diploid crossings are possible, they are generally less stable compared to their haploid counterparts and may result in tetraploid formations, which are usually not stable. This instability can significantly impact the efficacy and reliability of hybrid breeding outcomes in microbial applications. Crossing techniques follow a pattern illustrated in Figure 5a. First, two parents with complementary advantages are selected based on a comprehensive evaluation of the genetic diversity, metabolic activity, and enzyme production ability of the strains. One parent may have high-yield characteristics, while the other may have excellent metabolic pathways. Then, the selected parents are subjected to a human-controlled mating operation. In general, two parents are selected for a single cross or multiple crosses to increase genetic diversity and selection potential. The hybrid progeny after mating are screened. Screening for specific traits, such as enzyme production ability, tolerance, and tolerance, is usually used. Through screening, strains with excellent traits can be obtained. To further improve the traits of the strains, iterative hybridization and screening can be performed. Through multiple crosses and selections, the target characteristics can be gradually fixed, and stable strains can be developed. Munekazu et al. [124] found that the hybridization of the cryophilic wine yeasts *Saccharomyces bayanus* YM-84 and YM-126 with the mesophilic wine yeast *Saccharomyces cerevisiae* OC-2 had better fermentation performance than that of the mesophilic wine yeast; the malic acids and flavors produced by the hybrids were better than those produced by the mesophilic wine yeast. The content of compounds such as higher alcohols and isoamyl acetate is higher. Shinohara et al. [125] used thirty-one strains of *Saccharomyces cerevisiae* for hybridization. The results showed that the hybrids RIFT 1046, 1057, and 1065 could produce high concentrations of aromatic esters.

### 3.4. Protoplast Fusion Technique

Protoplast fusion technology is a technology in which the plastids of two or more cells are merged into a stable recombinant with the genetic characteristics of the parents [126,127,128]. The process of protoplast fusion is shown in Figure 5b. Protoplast fusion technology can break through the natural barrier of the cell wall and achieve long-distance genetic recombination; multiple recombinations have already been realized. Therefore, strains with better fermentation characteristics can be used as parents to screen for strains with even better fermentation characteristics through protoplast fusion. Xin et al. [129] used the fermenting strain Q, commonly used by beer manufacturers, for protoplast fusion with the haploids of strain L to improve the ethanol tolerance of strain Q while maintaining its fermentation performance. Wang et al. [130] used *S. cerevisiae* and *Candida ethanolica* as parents, and the alcohol content of the cider made from the stable fusion strains was 2.0–5.2%, which was far lower than that of the parental strains. In particular, the recombinant fusion yeast R6 was effective in producing high-quality, low-alcohol ciders. In the manufacturing of Chinese yellow wine, protoplast fusion technology has been used to breed yeast diploid hybrids with excellent wine-making characteristics, and the flavor characteristics of Chinese yellow wine were improved by using yeast diploid hybrids, leading to better ethanol tolerance and fermentability of the diploid parents [131].

### 3.5. Genetic Engineering Techniques

**Figure 6:**
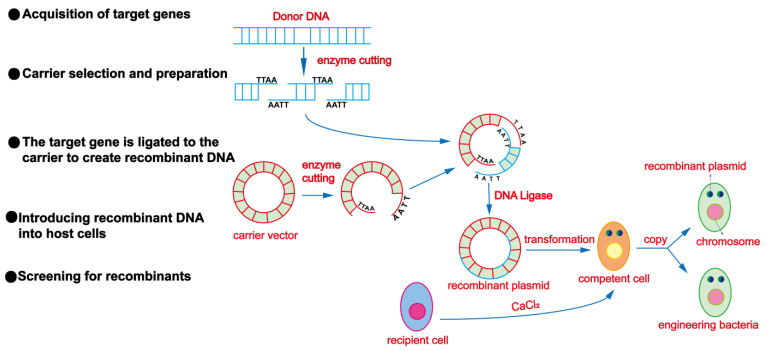
General process of genetic engineering techniques.

The core of genetic engineering techniques is the introduction of exogenous genes into organisms to achieve specific goals. These exogenous genes can be from other individuals of the same species, from a different species, or even from synthetic genes. After introducing exogenous genes into the target organisms, scientists can regulate the expression of these genes, thereby changing the traits of the organisms [132,133]. The general process of genetic engineering techniques is shown in Figure 6.

Many studies have shown that esters are an important component affecting the fruity aroma of wine. The enzyme accumulation of related esters during the fermentation process is the result of the balance between esterase synthesis and hydrolysis reactions, as well as the synthesis of alcohol acetyltransferase [134,135]. Alcohol acetyltransferase is an enzyme containing a sulfhydryl group that catalyzes the formation of esters between higher alcohols and acetyl-CoA. In *S. cerevisiae*, the ATF1 and ATF2 genes are the structural genes of the first group of acetyltransferases, encoding Alcohol Acetyltransferase I and Alcohol Acetyltransferase II, respectively [136]. Studies have shown that *S. cerevisiae* has one ATF1 gene, while beer yeast has one ATF1 and a homologous gene, Lg-ATF1 [137]. Yeast carrying multiple ATF1 genes or Lg-ATF1 genes had higher alcohol acetyltransferase activity and produced more acetates than yeast with only one ATF1 gene. The content of isoamyl acetate increased 27-fold, and the content of ethyl acetate increased 9-fold [138]. Lilly et al. [135] used PGK1 as a promoter and terminator and expressed the ATF1 gene in three commercial wine yeast strains. The contents of ethyl acetate, isoamyl acetate, and phenylethyl acetate in the products were all increased several-fold. Only slight changes in other esters. The gene of the edited protein in one strain is heterologously expressed in another strain with a stronger secretion ability, and the final target protein is obtained through isolation and purification, which can also achieve mass production with a high expression of specific enzymes. Kang et al. [139] cloned the prolyl endopeptidase gene from *Aspergillus oryzae* (AO-PEP) and expressed it in *Pichia pastoris*. After high-density batch fermentation, the AO-PEP activity reached 1130 U/L. The addition of AO-PEP in the fermentation stage can effectively reduce the turbidity of beer, improve the abiotic stability of the beer, and reduce the operational cost of adding polyvinylpolypyrrolidone to reduce turbidity.

### 3.6. Genome Shuffling Technology

**Figure 7:**
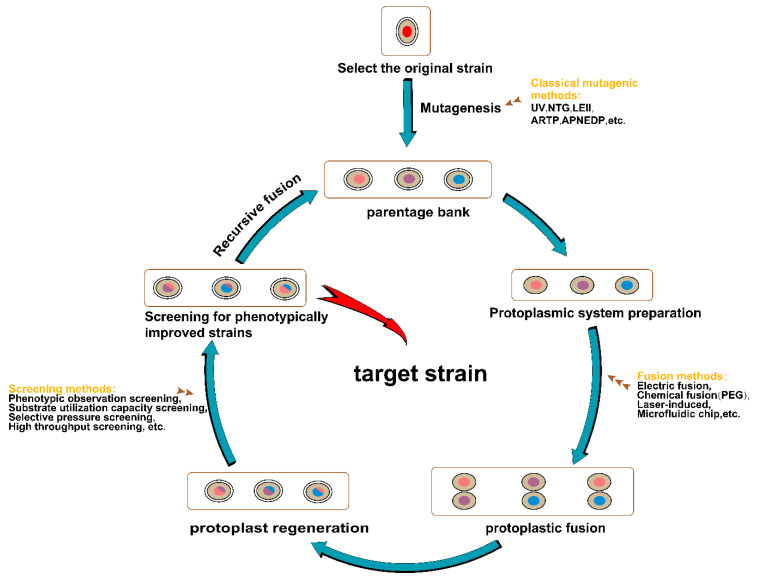
General process of genome shuffling technique.

Genome shuffling technology is a new technology for strain selection based on protoplast fusion technology [140,141]. Starting from one original parental strain, classic mutagenesis breeding methods were used to obtain multiple strains with significantly improved phenotypes, and a library of mutant candidate strains was constructed. This library was used for the direct parental strains for the first round of multiparental fusions and then further investigated. The whole genome was randomly recombined to obtain the first-generation fusion strains. Afterwards, strains with even further enhanced phenotypes were selected and used as the direct parents for the next round of fusions. Multiple rounds of multiparental strain fusions have been performed in this way, and targets with improved traits have been screened in the obtained mutant library [142]. In the breeding of wine-making microorganisms, genome shuffling technology can be applied to improve microorganisms, such as yeasts, to increase their efficiencies and yields in the alcohol fermentation process and improve the quality of wines. Shi et al. [143] performed three rounds of genome shuffling of *S. cerevisiae*. The improved strain SM-3 could grow on plate media at 55 °C; the heat tolerance, ethanol tolerance, and ethanol production of strain SM-3 were all improved; temperature is the limiting factor for the fermentation and production of *S. cerevisiae*. Snoek et al. [144] performed three rounds of genome shuffling on the parental strain of *S. cerevisiae* to eliminate improved strains with lower ethanol tolerance and obtain an improved *S. cerevisiae* strain with maximum ethanol production and a 7% increase in ethanol tolerance. Jetti et al. [145] used *S. cerevisiae* and *Pichia stipitis* for genome shuffling and obtained an improved strain, SP2-18, with a significantly higher substrate utilization rate than the parental strain. This strain could utilize xylose and had a high ethanol tolerance. Acceptance increased by 1.14 times. The general workflow of the genome shuffling technology is shown in Figure 7, which includes three parts: the construction of the parental library, the recursive fusion of protoplasts, and the screening of the target phenotype. The commonly used methods for microbial screening are compared, and their roles and method characteristics are shown in Table 2.

## 4. Application of Strain Selection Technology in Improving the Quality of Alcoholic Beverages

The primary task of strain selection technology is to screen microbial strains suitable for wine making. Through the collection and separation of samples from different sources, as well as the fermentation substrate screening method and the physiological characteristics screening method, microorganisms with good wine-making performance can be rapidly identified. The strain selection technology also includes the improvement of traditional vinification strains. Traditional strains can have better brewing performance through technical means such as genetic mutation or gene recombination. For example, genetic engineering technology can enhance the tolerance of strains to temperature, pH, alcohol concentration, and other conditions, thereby improving the stability and yield of the wine-making process. To broaden the strain resources of wine-making microorganisms, the introduction of new strains can also be considered. Through the collection and screening of samples from around the world, these new strains may have better fermentation efficiency and higher yields or bring new flavors and tastes. Genetic engineering technology has also been widely used in the breeding of wine-making microorganisms abroad. The gene editing and transformation of wine-making microbes can result in better brewing performance. For example, the introduction of improved yeast genes can improve fermentation efficiency and tolerance, and gene transfer technology can be used to introduce new functional genes into cerevisiae microorganisms, thus endowing them with new characteristics and functions. Table 3 lists some studies on selective breeding of alcoholic beverage microorganisms.

When it comes to sensory analysis of wine, the most important aspect to consider is its flavor characteristics. The proportion of flavor characteristics in the sensory evaluation of wine is more than 70%. For producers and dealers, the flavor quality of alcoholic beverages directly determines their economic value. For consumers, flavor characteristics greatly affect their judgment of the product’s quality. The metabolism of yeast is closely related to the formation of aroma-generating substances in wine. The selection of a fine, yellow wine yeast plays an important role in improving the aroma quality of yellow wine. Table 4 lists some of the studies on how to improve the flavor quality of different alcoholic beverage products. As shown in Table 4, the selection of a brewing microorganism with excellent performance can not only significantly improve the efficiency of alcoholic beverage production, but is also expected to increase the diversity of flavors in alcoholic beverages, thereby producing differentiated new wines with different styles.

## 5. Conclusions and Outlook

The microbial diversity of alcoholic beverages has a major influence on the flavor and quality of wine. Different kinds of microorganisms take part in the fermentation process of brewing wine, each playing its own role in the brewing process. The interaction and evolution between these microorganisms can produce beneficial substances while also potentially producing harmful substances that can reduce the quality of the wine. By exploring the microbial diversity in alcoholic beverages, we can understand its relationship with the quality and safety of these beverages. The strain selection technology can also help the brewing industry to obtain more microbial resources in wine making, enrich the diversity of microorganisms in wine making, improve the quality and yield of wine making, and reduce production costs and environmental pollution. However, the genetic stability of crossing, fusing, or genetic engineering is an important consideration. Complete stability assurance requires continuous monitoring and screening processes, which are directly related to the success rate of gene transfer and the quality of biological products. In addition, fermentation volumes have often been very low, meaning that although they may show positive effects under laboratory conditions, large-scale experiments may not yield the same positive results. The current research literature generally only shows test results for volatile chemicals and does not conduct blind wine tasting tests, so it is not possible to fully understand consumer acceptance of these products. Proper scientific investigation requires a deeper exploration of these key aspects. This paper summarizes the characteristics of microbial diversity in the world’s three major brewing alcohols and the selection technologies applicable to microorganisms in different wines. The goal is to better control and guide the wine-making process, increase flavor diversity, and ensure the continuation and development of wine-making culture. 

In current wine-making breeding work, physical mutagenesis, chemical mutagenesis, protoplast fusion, and other methods are widely used. Although these methods can achieve breeding goals to a certain extent, the huge screening work and uncertainty of breeding direction make progress slow. Improving the simplicity of mutation screening and the probability of a positive mutation in breeding is an important research topic for improving these methods. From current development trends, the use of gene-editing technology is the most direct and effective way to genetically modify the genetic material of strains. Genetic engineering breeding fundamentally excavates the synthesis mechanism of enzymes or metabolites at the molecular level, allowing for better guidance in selecting and breeding excellent strains in the future. Additionally, strain selection technology can be combined with advanced decoding technology that provides a deeper understanding of the genetic mechanisms and metabolic characteristics of microorganisms, providing a more accurate theoretical basis for selecting and applying alcoholic beverage microorganisms.

## Figures and Tables

**Table 1:** Dominant microorganisms in some typical alcoholic beverage varieties.

Alcoholic Beverage		Types	Dominant Microorganisms	Sensorial Properties	References
Yellow wine		Semi-dry Shaoxing Yellow Wine	*Bacillus*, *Lactobacillus*, *Leuconostoc*, *Lactococcus*, *Thermoactinomyces*	It has a pronounced rice aroma and light floral and fruity notes, as well as a distinctive earthiness from the fermentation process.	[36,42]
		CMQ (from Chongming, Shanghai)	*Pantoea*, *Bacillus*, *Rhizopus*, *Candida*	Soft, full-bodied, malty, and fruity on the palate.	[92]
		NBQ (from Ningbo, Zhejiang)	*Pediococcus*, *Lactobacillus*, *Acetobacter*, *Weissella*, *Bacillus*, *Rhizopus*, *Candida*, *Aspergillus*	Pale golden or orange-yellow in color, with a strong wheat and yeast aroma.	
		YCQ (from Yichang, Hubei)	*Pediococcus*, *Lactobacillus*, *Leuconostoc*, *Weissella*, *Lactococcus*, *Ochrobactrum*, *Rhizopus*, *Mucor*	Long aftertaste, often with a light sweetness in the aftertaste, refreshing taste.	
		Hong Qu glutinous yellow wine	*Bacillus*, *ginsengihumi*, *Pantoea* sp., *Elizabethkingia* sp., *Streptococcus* sp.	Reddish-brown, usually sweeter, with a distinctive hong qu aroma.	[93]
		Black glutinous yellow wine	*Pediococcus*, *Leuconostoc*, *Rhizopus*, *Saccharomycopsis*	Black or purple-black in color, with a sweet and sour taste and a complex aroma.	[94]
Wine	Red wine	Cabernet Sauvignon	*Pantoea*, *Lactobacillus*, *Rhodococcus*, *Fructobacillus*, and *Komagataeibacter*	Red wines typically have flavors of dark fruits like blackberry, cherry, and plum. They may also exhibit notes of chocolate, tobacco, and leather. Aromas can include earthy tones, spices, and sometimes a smoky character.	[95,96]
		Merlot	*Starmerella*, *Kazachstania*		[97,98]
		Shiraz	*Pseudomonas*, *Alternaria* sp.		[99]
		Pinot Noir	*Bacillus*		[99]
	White wine	Chardonnay	*Pseudomonas*, *Bacillus*, *Leuconostoc*, *Erwinia*	White wines usually have lighter, fresher flavors such as apples, pears, citrus, and tropical fruits. Floral and mineral aromas are also common.	[99,100]
		Riesling	*Pseudomonas*		[99]
		Sauvignon Blanc	/		[101,102]
	Sparkling wine	Champagne	/	Sparkling wine flavors often include green apple, pear, citrus, and sometimes toasty or nutty notes in aged varieties.	[103]
		Prosecco	/		[104]
	Dessert Wine	Noble Rot Wine	*Botrytis cinerea*	Dessert wine with flavors of dried fruit, honey, caramel, and nuts.	[105,106]
		Ice Wine	*Hanseniaspora uvarum*, *Metschnikowia fructicola*, *Saccharomyces cerevisiae*, *Lactococcus lactis and Leuconostoc* spp.		[107]
	Fortified Wine	Sherry	/	Fortified wines have a high alcohol content and intense flavors, including nutty, sweet, or spicy.	[108]
		Port	/		[109]
Beer		Lager beer, Pilsner beer	lager yeast	With a smooth and refreshing flavor.	[110]
		Ales, stouts, and porters	ale yeast	Often rich and complex with fruity, malty flavors, etc.	[111]
		Belgian Lambic Beer	*Saccharomyces cerevisiae*, *Lactobacillus*	With a distinctive sour and fruity flavor.	[110,112]

/: not found.

**Table 2:** Comparison of commonly used strain selection and breeding techniques.

Methods		Roles	Characteristics
Natural selection		In the natural environment, strains with better tolerance and adaptability are screened and bred through the process of natural selection, based on the genetic variation and adaptability of the species, without human intervention.	The presence of stochastic and temporal evolution with environmental dependence.
Crossing techniques		By crossing two different strains or lines of bacteria and yeast, their affinities and genetic variations are utilized to produce progeny strains with superior traits.	Can increase the genetic diversity of strains, be more stringent in the operation of tests and the choice of instruments, and have a high efficiency of selection and breeding.
Physical mutagenesis	UV mutagenesis	UV light causes base transitions, inversions, shifted mutations, or deletions, which can lead to mutagenesis of the strain.	Classic method, good results, simple equipment, and easy operation.
	Laser irradiation mutagenesis	When lasers irradiate organisms, their energy is directly or indirectly absorbed by biomolecules, which can lead to molecular stimulation of photodissociation, catabolism, and free radical reactions in biomolecules, resulting in aberrations in DNA molecules or chromosomes.	High energy density, relatively concentrated, good monochromaticity, and directionality; genetic mutation upon mutagenesis.
	Microwave mutagenesis	Can stimulate rapid vibration of polar molecules (e.g., water, proteins, nucleotides, fats, and carbohydrates), disrupting the hydrogen bonding and base accumulation of DNA molecules and leading to changes in the structure of the DNA, resulting in genetic variation.	Simple equipment, low cost, easy method, safe operation, and good mutation effect.
	High Static Pressure Mutagenesis	High hydrostatic pressure is a special processing technology for materials using hydrostatic pressure of more than 100MPa. It can not only change the volume, morphology, and cellular composition of microbial cells, but also alter the nucleic acid structure of microorganisms and their biological functions and gene expression.	Simple method, simple equipment, and good mutagenic effect.
	Ultrasound mutagenesis	Under the action of sound waves, the tiny bubbles in the liquid will oscillate, expand, contract, and even collapse. Cavitation bubble adiabatic contraction leads to the collapse of the moment, presenting more than 5000 °C temperatures and thousands of atmospheres of pressure, accompanied by powerful shock waves or jet streams, enough to change the cell wall membrane structure and cause the exchange of substances inside and outside the cells, and even mutation.	Simple equipment, safe operation, simple operation methods, and a higher mutation rate for mutagenesis.
Chemical mutagenesis		Techniques for selecting new mutagenic strains by inducing genetic variation in microorganisms using chemical mutagens	Operation of toxic and hazardous chemical substances for personal and environmental safety.
Protoplast fusion		The technique of artificially fusing two protoplasts with different genetic characteristics to obtain stable recombinants with parental genetic characteristics, and strains with good fermentation performance can be used directly as parental strains for the fermentation performance required for protoplast fusion breeding.	Overcoming the deficiencies of distant integration, simplicity of operation, integrity of genetic information, and a high frequency of recombination.
Genetic engineering techniques		Biogenetic traits targeted and directly modified at the DNA molecular level	Can lead to changes in the species’ orientation and relatively short selection cycles.
Genome shuffling		Through recursive recombination at the genomic level, the targeted evolution of the entire organism is efficiently realized, breaking through the limitations of traditional microbial strain improvement methods [146,147,148].	Phenotypic improvement of microbial strains can be accomplished by modifying the whole genome of multiple parents, without the need to know much about the genetic background of the modified strains.

**Table 3:** Studies on the selective breeding of certain alcoholic beverage microorganisms.

Field	Work	Results	References
Wine	Spore hybridization	Improvement of fermentation efficiency and SO_2_ tolerance	[149]
Wine	Insertion of the CUP1 gene at multiple loci to improve copper tolerance	Enhances antibacterial resistance	[150]
Wine	Adaptive evolution screening of novel wine yeast strains with improved characteristics	Enhances glycerol production; improves the taste of wine	[151]
Wine	Selection of yeast from orchard soil	High ethanol tolerance and improved fermentation performance	[152]
Beer	Mutagenesis screening of 2-DOG-resistant yeast	Improves the utilization and fermentation efficiency of polysaccharides	[153]
Shochu	Screening of trichothecene-resistant yeast	Improves fermentation efficiency	[154]
Sake	Fusion of sake yeast and beer yeast	Accelerated fermentation rate, high ester yield, and hypertonic resistance	[155]
Yellow wine	Removal of transcription regulators.	Low production of urea and ethyl carbamate	[49]
Yellow wine	Overexpression of ATF2 in industrial yellow wine yeast strain (RY1) by homologous recombination	Increases the acetate ester	[156]
Fruit wine	Protoplast fusion using *Saccharomyces cerevisiae* and *Candida ethanolica* as starting strains	Obtaining a new fusion yeast for aroma production and ethanol reduction	[130]
Rice wine	*Saccharomyces cerevisiae* hybrids created by directed evolution and protoplast fusion strategies	Screening of *Saccharomyces cerevisiae* with high fermentation efficiency and high stress resistance	[157,158]

**Table 4:** Some studies on how to improve the flavor quality of different types of alcoholic beverages.

Brewed Wine	Main Raw Material	Ways to Improve Flavor Quality	References
Sake	Rice	Breeding of sake yeast with high production of isoamyl acetate or ethyl hexanoate	[159,160,161]
Beer	Malt	Breeding of aroma-producing beer yeast	[162,163]
Fruit wine	Juice	Selection of yeast strains by protoplast fusion to regulate flavor substance production	[164,165]
Wine	Grapes	Selection of aroma-producing yeast by interspecific crossing	[11,166,167]
Yellow wine	Grain	Enrichment of microbial diversity in yellow wine brewing and improvement of brewing process	[15,168,169]

## Data Availability

No new data were created or analyzed in this study. Data sharing is not applicable to this article.

## References

[1] Patel P., Butani K., Kumar A., Singh S., Prajapati B.G. (2023). Effects of Fermented Food Consumption on Non-Communicable Diseases. Foods.

[2] Marco M.L., Heeney D., Binda S., Cifelli C.J., Cotter P.D., Foligné B., Gänzle M., Kort R., Pasin G., Pihlanto A. (2017). Health benefits of fermented foods: Microbiota and beyond. Curr. Opin. Biotechnol..

[3] Chen T., Qi X., Chen M., Lu D., Chen B. (2019). Discrimination of Chinese yellow wine from different origins based on flavor fingerprint. Acta Chromatogr..

[4] Zhou M.J., Bu T.T., Zheng J.X., Liu L., Yu S.F., Li S.S., Wu J.P. (2021). Peptides in Brewed Wines: Formation, Structure, and Function. J. Agric. Food Chem..

[5] Caspermeyer J. (2016). The Evolution of Beer. Mol. Biol. Evol..

[6] Hinojosa-Avila C.R., Garcia-Gamboa R., Chedraui-Urrea J.J.T., Garcia-Cayuela T. (2024). Exploring the potential of probiotic-enriched beer: Microorganisms, fermentation strategies, sensory attributes, and health implications. Food Res. Int..

[7] Wu Q., Li L.M., Xiang P., Zhang T., Peng L.X., Zou L., Li Q. (2023). Phages in Fermented Foods: Interactions and Applications. Fermentation.

[8] van Wyk N., Grossmann M., Wendland J., von Wallbrunn C., Pretorius I.S. (2019). The Whiff of Wine Yeast Innovation: Strategies for Enhancing Aroma Production by Yeast during Wine Fermentation. J. Agric. Food Chem..

[9] Mendes Ferreira A., Mendes-Faia A. (2020). The Role of Yeasts and Lactic Acid Bacteria on the Metabolism of Organic Acids during Winemaking. Foods.

[10] Munford A.R.G., Chaves R.D., Sant’Ana A.S. (2020). Inactivation kinetics of beer spoilage bacteria (*Lactobacillus brevis*, *Lactobacillus casei*, and *Pediococcus damnosus*) during acid washing of brewing yeast. Food Microbiol..

[11] Steensels J., Meersman E., Snoek T., Saels V., Verstrepen K.J. (2014). Large-scale selection and breeding to generate industrial yeasts with superior aroma production. Appl. Environ. Microbiol..

[12] Su J., Wang T., Wang Y., Li Y.Y., Li H. (2014). The use of lactic acid-producing, malic acid-producing, or malic acid-degrading yeast strains for acidity adjustment in the wine industry. Appl. Microbiol. Biotechnol..

[13] Tilloy V., Ortiz-Julien A., Dequin S. (2014). Reduction of ethanol yield and improvement of glycerol formation by adaptive evolution of the wine yeast *Saccharomyces cerevisiae* under hyperosmotic conditions. Appl. Environ. Microbiol..

[14] Su Y., Gamero A., Rodríguez M.E., Lopes C.A., Querol A., Guillamón J.M. (2019). Interspecific hybridisation among diverse Saccharomyces species: A combined biotechnological solution for low-temperature and nitrogen-limited wine fermentations. Int. J. Food Microbiol..

[15] Yang Y., Hu W., Xia Y., Mu Z., Tao L., Song X., Zhang H., Ni B., Ai L. (2020). Flavor Formation in Chinese Rice Wine (Huangjiu): Impacts of the Flavor-Active Microorganisms, Raw Materials, and Fermentation Technology. Front. Microbiol..

[16] Shuai H., Xiangzhao M., Pei L., Hong L., Zuyuan D., Ning L., Jichen H., Cuifang Q. (2013). Research into the functional components and antioxidant activities of North China rice wine (Ji Mo Lao Jiu). Food Sci. Nutr..

[17] Lv X.-C., Chen Z.-C., Jia R.-B., Liu Z.-B., Zhang W., Chen S.-J., Rao P.-F., Ni L. (2015). Microbial community structure and dynamics during the traditional brewing of Fuzhou Hong Qu glutinous rice wine as determined by culture-dependent and culture-independent techniques. Food Control.

[18] Wu Z., Xu E., Long J., Pan X., Xu X., Jin Z., Jiao A. (2016). Comparison between ATR-IR, Raman, concatenated ATR-IR and Raman spectroscopy for the determination of total antioxidant capacity and total phenolic content of Chinese rice wine. Food Chem..

[19] Lin H., Meng L., Sun Z., Sun S., Huang X., Lin N., Zhang J., Lu W., Yang Q., Chi J. (2021). Yellow Wine Polyphenolic Compound Protects Against Doxorubicin-Induced Cardiotoxicity by Modulating the Composition and Metabolic Function of the Gut Microbiota. Circ. Heart Fail..

[20] Yang J., Song J., Zhou J., Lin H., Wu Z., Liu N., Xie W., Guo H., Chi J. (2022). Functional components of Chinese rice wine can ameliorate diabetic cardiomyopathy through the modulation of autophagy, apoptosis, gut microbiota, and metabolites. Front. Cardiovasc. Med..

[21] Zhao C., Su W., Mu Y., Luo L., Zhao M., Qiu S., Su G., Jiang L. (2023). Effects of Jiuqu inoculating Rhizopus oryzae Q303 and *Saccharomyces cerevisiae* on chemical components and microbiota during black glutinous rice wine fermentation. Int. J. Food Microbiol..

[22] Tian S., Zeng W., Fang F., Zhou J., Du G. (2022). The microbiome of Chinese rice wine (Huangjiu). Curr. Res. Food Sci..

[23] Londoño-Hernández L., Ramírez-Toro C., Ruiz H.A., Ascacio-Valdés J.A., Aguilar-Gonzalez M.A., Rodríguez-Herrera R., Aguilar C.N. (2017). Rhizopus oryzae—Ancient microbial resource with importance in modern food industry. Int. J. Food Microbiol..

[24] Chen T., Wu F., Guo J., Ye M., Hu H., Guo J., Liu X. (2020). Effects of glutinous rice protein components on the volatile substances and sensory properties of Chinese rice wine. J. Sci. Food Agric..

[25] Chang P.K., Scharfenstein L.L., Solorzano C.D., Abbas H.K., Hua S.S., Jones W.A., Zablotowicz R.M. (2015). High sequence variations in the region containing genes encoding a cellular morphogenesis protein and the repressor of sexual development help to reveal origins of Aspergillus oryzae. Int. J. Food Microbiol..

[26] Rui Y., Wan P., Chen G., Xie M., Sun Y., Zeng X., Liu Z. (2019). Analysis of bacterial and fungal communities by Illumina MiSeq platforms and characterization of Aspergillus cristatus in Fuzhuan brick tea. LWT.

[27] Liu S., Yang L., Zhou Y., He S., Li J., Sun H., Yao S., Xu S. (2019). Effect of mixed moulds starters on volatile flavor compounds in rice wine. LWT.

[28] Liu Y., Yue Z., Jinglei L., Shan L., Shudong H., Hanju S., Shengfei Y., Shangying X. (2021). Effect of enzymes addition on the fermentation of Chinese rice wine using defined fungal starter. LWT.

[29] Yang Y., Zhong H., Yang T., Lan C., Zhu H. (2021). Characterization of the key aroma compounds of a sweet rice alcoholic beverage fermented with Saccharomycopsis fibuligera. J. Food Sci. Technol..

[30] Jolly N.P., Varela C., Pretorius I.S. (2014). Not your ordinary yeast: Non-Saccharomyces yeasts in wine production uncovered. FEMS Yeast Res..

[31] Pretorius I.S. (2000). Tailoring wine yeast for the new millennium: Novel approaches to the ancient art of winemaking. Yeast.

[32] Pretorius I.S., Bauer F.F. (2002). Meeting the consumer challenge through genetically customized wine-yeast strains. Trends Biotechnol..

[33] Pretorius I.S., Curtin C.D., Chambers P.J. (2012). The winemaker’s bug: From ancient wisdom to opening new vistas with frontier yeast science. Bioeng. Bugs.

[34] Huang Z.R., Hong J.L., Xu J.X., Li L., Guo W.L., Pan Y.Y., Chen S.J., Bai W.D., Rao P.F., Ni L. (2018). Exploring core functional microbiota responsible for the production of volatile flavour during the traditional brewing of Wuyi Hong Qu glutinous rice wine. Food Microbiol..

[35] Canonico L., Solomon M., Comitini F., Ciani M., Varela C. (2019). Volatile profile of reduced alcohol wines fermented with selected non-Saccharomyces yeasts under different aeration conditions. Food Microbiol..

[36] Liu S.P., Mao J., Liu Y.Y., Meng X.Y., Ji Z.W., Zhou Z.L., Ai-lati A. (2015). Bacterial succession and the dynamics of volatile compounds during the fermentation of Chinese rice wine from Shaoxing region. World J. Microbiol. Biotechnol..

[37] Lv X.-C., Huang R.-L., Chen F., Zhang W., Rao P.-F., Ni L. (2013). Bacterial community dynamics during the traditional brewing of Wuyi Hong Qu glutinous rice wine as determined by culture-independent methods. Food Control.

[38] Cai H., Zhang T., Zhang Q., Luo J., Cai C., Mao J. (2018). Microbial diversity and chemical analysis of the starters used in traditional Chinese sweet rice wine. Food Microbiol..

[39] Liu H., Sun B. (2018). Effect of Fermentation Processing on the Flavor of Baijiu. J. Agric. Food Chem..

[40] Zhang J., Fang L., Huang X., Ding Z., Wang C. (2022). Evolution of polyphenolic, anthocyanin, and organic acid components during coinoculation fermentation (simultaneous inoculation of LAB and yeast) and sequential fermentation of blueberry wine. J. Food Sci..

[41] Li P.P., Su R., Wang Q., Liu K.Y., Yang H., Du W., Li Z.A., Chen S., Xu B., Yang W. (2022). Comparison of fungal communities and nonvolatile flavor components in black Huangjiu formed using different inoculation fermentation methods. Front. Microbiol..

[42] Liu S.P., Yu J.X., Wei X.L., Ji Z.W., Zhou Z.L., Meng X.Y., Mao J. (2016). Sequencing-based screening of functional microorganism to decrease the formation of biogenic amines in Chinese rice wine. Food Control.

[43] Lv X.C., Jiang Y.J., Liu J., Guo W.L., Liu Z.B., Zhang W., Rao P.F., Ni L. (2017). Evaluation of different PCR primers for denaturing gradient gel electrophoresis (DGGE) analysis of fungal community structure in traditional fermentation starters used for Hong Qu glutinous rice wine. Int. J. Food Microbiol..

[44] Wang P., Mao J., Meng X., Li X., Liu Y., Feng H. (2014). Changes in flavour characteristics and bacterial diversity during the traditional fermentation of Chinese rice wines from Shaoxing region. Food Control.

[45] Qingtao L., Xinhui Y., Qixing L., Jianghua L., Fang F., Guocheng D., Zhen K. (2018). Molecular Engineering of Bacillus paralicheniformis Acid Urease To Degrade Urea and Ethyl Carbamate in Model Chinese Rice Wine. J. Agric. Food Chem..

[46] Xiaole X., Yi L., Qingwen Z., Yang H., Bin Z. (2018). Mixed Starter Culture Regulates Biogenic Amines Formation via Decarboxylation and Transamination during Chinese Rice Wine Fermentation. J. Agric. Food Chem..

[47] Wu P., Cai C., Shen X., Wang L., Zhang J., Tan Y., Jiang W., Pan X. (2014). Formation of ethyl carbamate and changes during fermentation and storage of yellow rice wine. Food Chem..

[48] Shalamitskiy M.Y., Tanashchuk T.N., Cherviak S.N., Vasyagin E.A., Ravin N.V., Mardanov A.V. (2023). Ethyl Carbamate in Fermented Food Products: Sources of Appearance, Hazards and Methods for Reducing Its Content. Foods.

[49] Wei T.Y., Jiao Z.H., Hu J.J., Lou H.H., Chen Q.H. (2020). Chinese Yellow Rice Wine Processing with Reduced Ethyl Carbamate Formation by Deleting Transcriptional Regulator Dal80p in *Saccharomyces cerevisiae*. Molecules.

[50] Pashangeh S., Berizi E., Majlesi M., Ghaderi S., Nizet V., Dahesh S. (2022). Effect of eliminating hdcA gene of Staphylococcus epidermidis TYH1 on Histamine production. Iran. J. Microbiol..

[51] Rodríguez-Lucas C., Ladero V. (2023). Enterococcal Phages: Food and Health Applications. Antibiotics.

[52] Del Rio B., Ladero V., Redruello B., Linares D.M., Fernandez M., Martin M.C., Alvarez M.A. (2015). Lactose-mediated carbon catabolite repression of putrescine production in dairy Lactococcus lactis is strain dependent. Food Microbiol..

[53] Luo Y., Huang Y., Xu R.X., Qian B., Zhou J.W., Xia X.L. (2020). Primary and Secondary Succession Mediate the Accumulation of Biogenic Amines during Industrial Semidry Chinese Rice Wine Fermentation. Appl. Environ. Microbiol..

[54] Wojtowicz J.S. (2023). Long-Term Health Outcomes of Regular, Moderate Red Wine Consumption. Cureus.

[55] Perrone B., Giacosa S., Rolle L., Cocolin L., Rantsiou K. (2013). Investigation of the dominance behavior of *Saccharomyces cerevisiae* strains during wine fermentation. Int. J. Food Microbiol..

[56] Cappello M.S., Zapparoli G., Logrieco A., Bartowsky E.J. (2017). Linking wine lactic acid bacteria diversity with wine aroma and flavour. Int. J. Food Microbiol..

[57] Mateo E., Torija M.J., Mas A., Bartowsky E.J. (2014). Acetic acid bacteria isolated from grapes of South Australian vineyards. Int. J. Food Microbiol..

[58] Testa B., Lombardi S.J., Tremonte P., Succi M., Tipaldi L., Pannella G., Sorrentino E., Iorizzo M., Coppola R. (2014). Biodiversity of Lactobacillus plantarum from traditional Italian wines. World J. Microbiol. Biotechnol..

[59] Zhang S.W., Chen X., Zhong Q.D., Zhuang X.L., Bai Z.H. (2019). Microbial Community Analyses Associated with Nine Varieties of Wine Grape Carposphere Based on High-Throughput Sequencing. Microorganisms.

[60] Maicas S. (2001). The use of alternative technologies to develop malolactic fermentation in wine. Appl. Microbiol. Biotechnol..

[61] Sumby K.M., Bartle L., Grbin P.R., Jiranek V. (2019). Measures to improve wine malolactic fermentation. Appl. Microbiol. Biotechnol..

[62] Sun J., Ge Y., Gu X., Li R., Ma W., Jin G. (2022). Identification and Characterization of Malolactic Bacteria Isolated from the Eastern Foothills of Helan Mountain in China. Foods.

[63] Michlmayr H., Nauer S., Brandes W., Schümann C., Kulbe K.D., del Hierro A.M., Eder R. (2012). Release of wine monoterpenes from natural precursors by glycosidases from *Oenococcus oeni*. Food Chem..

[64] Gagné S., Lucas P.M., Perello M.C., Claisse O., Lonvaud-Funel A., de Revel G. (2011). Variety and variability of glycosidase activities in an *Oenococcus oeni* strain collection tested with synthetic and natural substrates. J. Appl. Microbiol..

[65] Ferner M.J., Müller G., Schumann C., Shaikh Y., Kmapeis P., Ulber R., Raddatz H. (2018). Immobilisation of glycosidases from commercial preparation on magnetic beads. Part 2: Aroma enhancement in wine using immobilised glycosidases. Vitis.

[66] Tavernini L., Aburto C., Romero O., Illanes A., Wilson L. (2021). Encapsulation of Combi-CLEAs of Glycosidases in Alginate Beads and Polyvinyl Alcohol for Wine Aroma Enhancement. Catalysts.

[67] Ahumada K., Urrutia P., Illanes A., Wilson L. (2015). Production of combi-CLEAs of glycosidases utilized for aroma enhancement in wine. Food Bioprod. Process..

[68] González-Pombo P., Fariña L., Carrau F., Batista-Viera F., Brena B.M. (2014). Aroma enhancement in wines using co-immobilized *Aspergillus niger* glycosidases. Food Chem..

[69] Bartowsky E.J., Henschke P.A. (2008). Acetic acid bacteria spoilage of bottled red wine—A review. Int. J. Food Microbiol..

[70] Bartowsky E.J., Xia D., Gibson R.L., Fleet G.H., Henschke P.A. (2003). Spoilage of bottled red wine by acetic acid bacteria. Lett. Appl. Microbiol..

[71] Gupta A., Singh V.K., Qazi G.N., Kumar A. (2001). Gluconobacter oxydans: Its biotechnological applications. J. Mol. Microbiol. Biotechnol..

[72] Nurgel C., Pickering G.J., Inglis D.L. (2004). Sensory and chemical characteristics of Canadian ice wines. J. Sci. Food Agric..

[73] Izquierdo-Canas P.M., Lopez-Martin R., Garcia-Romero E., Gonzalez-Arenzana L., Minguez-Sanz S., Chatonnet P., Palacios-Garcia A., Puig-Pujol A. (2018). Effect of kaolin silver complex on the control of populations of Brettanomyces and acetic acid bacteria in wine. J. Food Sci. Technol..

[74] Bae S., Fleet G.H., Heard G.M. (2004). Occurrence and significance of Bacillus thuringiensis on wine grapes. Int. J. Food Microbiol..

[75] Hranilovic A., Gambetta J.M., Jeffery D.W., Grbin P.R., Jiranek V. (2020). Lower-alcohol wines produced by Metschnikowia pulcherrima and *Saccharomyces cerevisiae* co-fermentations: The effect of sequential inoculation timing. Int. J. Food Microbiol..

[76] Romano P., Fiore C., Paraggio M., Caruso M., Capece A. (2003). Function of yeast species and strains in wine flavour. Int. J. Food Microbiol..

[77] Oliveira E.S., Cardello H.M.A.B., Jeronimo E.M., Souza E.L.R., Serra G.E. (2005). The influence of different yeasts on the fermentation, composition and sensory quality of cachaca. World J. Microbiol. Biotechnol..

[78] Domizio P., Romani C., Lencioni L., Comitini F., Gobbi M., Mannazzu I., Ciani M. (2011). Outlining a future for non-Saccharomyces yeasts: Selection of putative spoilage wine strains to be used in association with *Saccharomyces cerevisiae* for grape juice fermentation. Int. J. Food Microbiol..

[79] Dutraive O., Benito S., Fritsch S., Beisert B., Patz C.D., Rauhut D. (2019). Effect of Sequential Inoculation with Non-*Saccharomyces* and *Saccharomyces* Yeasts on Riesling Wine Chemical Composition. Fermentation.

[80] Ge Q., Guo C.F., Zhang J., Yan Y., Zhao D.Q., Li C.H., Sun X.Y., Ma T.T., Yue T.L., Yuan Y.H. (2021). Effects of Simultaneous Co-Fermentation of Five Indigenous Non-Saccharomyces Strains with *S. cerevisiae* on Vidal Icewine Aroma Quality. Foods.

[81] Fedrizzi B., Caramia G., Cipriani M., Finato F., Simonato B., Tosi E., Zapparoli G. (2011). Changes in Wine Aroma Composition According to Botrytized Berry Percentage: A Preliminary Study on Amarone Wine. Food Technol. Biotechnol..

[82] Tosi E., Fedrizzi B., Azzolini M., Finato F., Simonato B., Zapparoli G. (2012). Effects of noble rot on must composition and aroma profile of Amarone wine produced by the traditional grape withering protocol. Food Chem..

[83] Bamforth C.W. (2017). Progress in Brewing Science and Beer Production. Annu. Rev. Chem. Biomol. Eng..

[84] Liu J., Cai G.L., Li X.M., Lu J. (2023). Development of a novel SRAP-SCAR marker for rapid identification of lager and ale types in brewer’s yeast. Mol. Biol. Rep..

[85] Kato T., Takahashi T. (2023). Studies on the Genetic Characteristics of the Brewing Yeasts Saccharomyces: A Review. J. Am. Soc. Brew. Chem..

[86] Pereira G.M.D., Ramos C.L., Galvao C., Dias E.S., Schwan R.F. (2010). Use of specific PCR primers to identify three important industrial species of *Saccharomyces* genus: *Saccharomyces cerevisiae*, *Saccharomyces bayanus* and *Saccharomyces pastorianus*. Lett. Appl. Microbiol..

[87] Monerawela C., Bond U. (2017). Recombination sites on hybrid chromosomes in *Saccharomyces pastorianus* share common sequence motifs and define a complex evolutionary relationship between group I and II lager yeasts. Fems Yeast Res..

[88] Suzuki K., Iijima K., Sakamoto K., Sami M., Yamashita H. (2006). A Review of Hop Resistance in Beer Spoilage Lactic Acid Bacteria. J. Inst. Brew..

[89] Hucker B., Christophersen M., Vriesekoop F. (2017). The influence of thiamine and riboflavin on various spoilage microorganisms commonly found in beer. J. Inst. Brew..

[90] Soto J.B., Fernandez-Franzon M., Ruiz M.J., Juan-Garcia A. (2014). Presence of Ochratoxin A (OTA) Mycotoxin in Alcoholic Drinks from Southern European Countries: Wine and Beer. J. Agric. Food Chem..

[91] Dysvik A., La Rosa S.L., Liland K.H., Myhrer K.S., Østlie H.M., De Rouck G., Rukke E.O., Westereng B., Wicklund T. (2020). Co-fermentation Involving *Saccharomyces cerevisiae* and Lactobacillus Species Tolerant to Brewing-Related Stress Factors for Controlled and Rapid Production of Sour Beer. Front. Microbiol..

[92] Chen L.H., Ren L.X., Li D.N., Ma X. (2021). Analysis of microbiomes in three traditional starters and volatile components of the Chinese rice wines. Food Sci. Biotechnol..

[93] Huang Z.R., Guo W.L., Zhou W.B., Li L., Xu J.X., Hong J.L., Liu H.P., Zeng F., Bai W.D., Liu B. (2019). Microbial communities and volatile metabolites in different traditional fermentation starters used for Hong Qu glutinous rice wine. Food Res. Int..

[94] Jiang L., Su W., Mu Y.C., Mu Y. (2020). Major Metabolites and Microbial Community of Fermented Black Glutinous Rice Wine with Different Starters. Front. Microbiol..

[95] Zhang Z., Zhang Q.C., Yang H., Sun L.J., Xia H.C., Sun W.J., Wang Z., Zhang J.X. (2022). Bacterial Communities Related to Aroma Formation during Spontaneous Fermentation of ‘Cabernet Sauvignon’ Wine in Ningxia, China. Foods.

[96] Pons A., Mouakka N., Deliere L., Crachereau J.C., Davidou L., Sauris P., Guilbault P., Darriet P. (2018). Impact of *Plasmopara viticola* infection of Merlot and Cabernet Sauvignon grapes on wine composition and flavor. Food Chem..

[97] Zhang L., Tao Y.S., Wen Y., Wang H. (2013). Aroma Evaluation of Young Chinese Merlot Wines with Denomination of Origin. S. Afr. J. Enol. Vitic..

[98] Ma Y.W., Peng S., Mi L., Li M., Jiang Z.Z., Wang J. (2023). Correlation between fungi and volatile compounds during different fermentation modes at the industrial scale of Merlot wines. Food Res. Int..

[99] Zhang S.W., Wang Y., Chen X., Cui B.J., Bai Z.H., Zhuang G.Q. (2020). Variety features differentiate microbiota in the grape leaves. Can. J. Microbiol..

[100] Leveau J., Tech J.J. (2011). Grapevine microbiomics: Bacterial diversity on grape leaves and berries revealed by high-throughput sequence analysis of 16S rRNA amplicons. Acta Hortic..

[101] Coetzee C., du Toit W.J. (2012). A comprehensive review on Sauvignon blanc aroma with a focus on certain positive volatile thiols. Food Res. Int..

[102] Lund C.M., Thompson M.K., Benkwitz F., Wohler M.W., Triggs C.M., Gardner R., Heymann H., Ncolau L. (2009). New Zealand Sauvignon blanc Distinct Flavor Characteristics: Sensory, Chemical, and Consumer Aspects. Am. J. Enol. Vitic..

[103] Schmidt C.V., Olsen K., Mouritsen O.G. (2021). Umami potential of fermented beverages: Sake, wine, champagne, and beer. Food Chem..

[104] Slaghenaufi D., Luzzini G., Borgato M., Boscaini A., Dal Cin A., Zandonà V., Ugliano M. (2023). Characterization of the Aroma Profile of Commercial Prosecco Sparkling Wines. Appl. Sci..

[105] Kallitsounakis G., Catarino S. (2020). An overview on botrytized wines. Ciência Técnica Vitivinícola.

[106] Li H., James A., Shen X.M., Wang Y.S. (2021). Roles of microbiota in the formation of botrytized grapes and wines. Cyta J. Food.

[107] Chen Y., Zhang W., Yi H., Wang B., Xiao J., Zhou X.Y., Xu J.K., Jiang L., Shi X.W. (2020). Microbial community composition and its role in volatile compound formation during the spontaneous fermentation of ice wine made from Vidal grapes. Process Biochem..

[108] Durán-Guerrero E., Castro-Mejías R., García-Moreno M.D., Rodríguez-Dodero M.D., Schwarz M., Guillén-Sánchez D. (2021). Aroma of Sherry Products: A Review. Foods.

[109] Prata-Sena M., Castro-Carvalho B.M., Nunes S., Amaral B., Silva P. (2018). The terroir of Port wine: Two hundred and sixty years of history. Food Chem..

[110] De Roos J., De Vuyst L. (2019). Microbial acidification, alcoholization, and aroma production during spontaneous lambic beer production. J. Sci. Food Agric..

[111] Bonatto D. (2021). The diversity of commercially available ale and lager yeast strains and the impact of brewer’s preferential yeast choice on the fermentative beer profiles. Food Res. Int..

[112] De Roos J., De Vuyst L. (2018). Acetic acid bacteria in fermented foods and beverages. Curr. Opin. Biotechnol..

[113] Vaughan A., O’Sullivan T., van Sinderen D. (2005). Enhancing the microbiological stability of malt and beer—A review. J. Inst. Brew..

[114] Vion C., Peltier E., Bernard M., Muro M., Marullo P. (2021). Marker Assisted Selection of Malic-Consuming *Saccharomyces cerevisiae* Strains for Winemaking. Efficiency and Limits of a QTL’s Driven Breeding Program. J. Fungi.

[115] Bellon J.R., Ford C.M., Borneman A.R., Chambers P.J. (2018). A Novel Approach to Isolating Improved Industrial Interspecific Wine Yeasts Using Chromosomal Mutations as Potential Markers for Increased Fitness. Front. Microbiol..

[116] Jung J.Y., Kang M.J., Hwang H.S., Baek K.R., Seo S.O. (2022). Reduction of Ethyl Carbamate in an Alcoholic Beverage by CRISPR/Cas9-Based Genome Editing of the Wild Yeast. Foods.

[117] Pérez-Coello M.S., Briones Pérez A.I., Ubeda Iranzo J.F., Martin Alvarez P.J. (1999). Characteristics of wines fermented with different *Saccharomyces cerevisiae* strains isolated from the La Mancha region. Food Microbiol..

[118] Zheng H., Meng K., Liu J., Lin Z., Peng Q., Xie G., Wu P., Elsheery N.I. (2022). Identification and expression of bifunctional acid urea-degrading enzyme/urethanase from *Enterobacter* sp. R-SYB082 and its application in degradation of ethyl carbamate in Chinese rice wine (Huangjiu). J. Sci. Food Agric..

[119] McCarthy T.C., Lalor E., Hanniffy O., Savage A.V., Tuohy M.G. (2005). Comparison of wild-type and UV-mutant beta-glucanase-producing strains of Talaromyces emersonii with potential in brewing applications. J. Ind. Microbiol. Biotechnol..

[120] Chandel A.K., Kapoor R.K., Singh A., Kuhad R.C. (2007). Detoxification of sugarcane bagasse hydrolysate improves ethanol production by Candida shehatae NCIM 3501. Bioresour. Technol..

[121] Laughery M.F., Plummer D.A., Wilson H.E., Vandenberg B.N., Mitchell D., Mieczkowski P.A., Roberts S.A., Wyrick J.J. (2023). Genome-wide maps of UVA and UVB mutagenesis in yeast reveal distinct causative lesions and mutational strand asymmetries. Genetics.

[122] Yi S., Zhang X., Li H.X., Du X.X., Liang S.W., Zhao X.H. (2018). Screening and Mutation of *Saccharomyces cerevisiae* UV-20 with a High Yield of Second Generation Bioethanol and High Tolerance of Temperature, Glucose and Ethanol. Ind. J. Microbiol..

[123] Yang T., Zhang S., Li L., Tian J., Li X., Pan Y. (2022). Screening and transcriptomic analysis of the ethanol-tolerant mutant *Saccharomyces cerevisiae* YN81 for high-gravity brewing. Front. Microbiol..

[124] Munekazu K. (1994). Fermentation characteristics of hybrids between the cryophilic wine yeast *Saccharomyces bayanus* and the mesophilic wine yeast *Saccharomyces cerevisiae*. J. Ferment. Bioeng..

[125] Shinohara T., Saito K., Yanagida F., Goto S. (1994). Selection and hybridization of wine yeasts for improved winemaking properties: Fermentation rate and aroma productivity. J. Ferment. Bioeng..

[126] Skała J., Kotylak Z. (1984). Protoplast fusion in *Saccharomyces cerevisiae*. Acta Microbiol. Pol..

[127] Morgan A.J. (1983). Yeast strain improvement by protoplast fusion and transformation. Experientia. Suppl..

[128] Kavanagh K., Walsh M., Whittaker P.A. (1991). Enhanced intraspecific protoplast fusion in yeast. FEMS Microbiol. Lett..

[129] Xin Y., Yang M., Yin H., Yang J. (2020). Improvement of Ethanol Tolerance by Inactive Protoplast Fusion in *Saccharomyces cerevisiae*. BioMed Res. Int..

[130] Wang Z., Xu K., Cai R., Yue T., Yuan Y., Gao Z. (2020). Construction of recombinant fusant yeasts for the production of cider with low alcohol and enhanced aroma. Eur. Food Res. Technol..

[131] Yang Y., Xia Y., Lin X., Wang G., Zhang H., Xiong Z., Yu H., Yu J., Ai L. (2018). Improvement of flavor profiles in Chinese rice wine by creating fermenting yeast with superior ethanol tolerance and fermentation activity. Food Res. Int..

[132] Kuzma J. (2016). Policy: Reboot the debate on genetic engineering. Nature.

[133] Liang Z., Zhi H., Fang Z., Zhang P. (2021). Genetic engineering of yeast, filamentous fungi and bacteria for terpene production and applications in food industry. Food Res. Int..

[134] Matthews A., Grbin P.R., Jiranek V. (2007). Biochemical characterisation of the esterase activities of wine lactic acid bacteria. Appl. Microbiol. Biotechnol..

[135] Lilly M., Lambrechts M.G., Pretorius I.S. (2000). Effect of increased yeast alcohol acetyltransferase activity on flavor profiles of wine and distillates. Appl. Environ. Microbiol..

[136] Mason A.B., Dufour J.P. (2000). Alcohol acetyltransferases and the significance of ester synthesis in yeast. Yeast.

[137] Fujii T., Nagasawa N., Iwamatsu A., Bogaki T., Tamai Y., Hamachi M. (1994). Molecular cloning, sequence analysis, and expression of the yeast alcohol acetyltransferase gene. Appl. Environ. Microbiol..

[138] Lilly M., Bauer F.F., Lambrechts M.G., Swiegers J.H., Cozzolino D., Pretorius I.S. (2006). The effect of increased yeast alcohol acetyltransferase and esterase activity on the flavour profiles of wine and distillates. Yeast.

[139] Kang C., Yu X.W., Xu Y. (2015). Cloning and expression of a novel prolyl endopeptidase from Aspergillus oryzae and its application in beer stabilization. J. Ind. Microbiol. Biotechnol..

[140] Stemmer W.P. (1994). DNA shuffling by random fragmentation and reassembly: In vitro recombination for molecular evolution. Proc. Natl. Acad. Sci. USA.

[141] Zhang Y.X., Perry K., Vinci V.A., Powell K., Stemmer W.P., del Cardayré S.B. (2002). Genome shuffling leads to rapid phenotypic improvement in bacteria. Nature.

[142] Gong J., Zheng H., Wu Z., Chen T., Zhao X. (2009). Genome shuffling: Progress and applications for phenotype improvement. Biotechnol. Adv..

[143] Shi D.J., Wang C.L., Wang K.M. (2009). Genome shuffling to improve thermotolerance, ethanol tolerance and ethanol productivity of *Saccharomyces cerevisiae*. J. Ind. Microbiol. Biotechnol..

[144] Snoek T., Picca Nicolino M., Van den Bremt S., Mertens S., Saels V., Verplaetse A., Steensels J., Verstrepen K.J. (2015). Large-scale robot-assisted genome shuffling yields industrial *Saccharomyces cerevisiae* yeasts with increased ethanol tolerance. Biotechnol. Biofuels.

[145] Jetti K.D., Gns R.R., Garlapati D., Nammi S.K. (2019). Improved ethanol productivity and ethanol tolerance through genome shuffling of *Saccharomyces cerevisiae* and Pichia stipitis. Int. Microbiol. Off. J. Span. Soc. Microbiol..

[146] Gu C., Wang G., Mai S., Wu P., Wu J., Wang G., Liu H., Zhang J. (2017). ARTP mutation and genome shuffling of ABE fermentation symbiotic system for improvement of butanol production. Appl. Microbiol. Biotechnol..

[147] Li S., Chen X., Dong C., Zhao F., Tang L., Mao Z. (2013). Combining genome shuffling and interspecific hybridization among Streptomyces improved ε-poly-L-lysine production. Appl. Biochem. Biotechnol..

[148] John R.P., Gangadharan D., Madhavan Nampoothiri K. (2008). Genome shuffling of *Lactobacillus delbrueckii* mutant and *Bacillus amyloliquefaciens* through protoplasmic fusion for L-lactic acid production from starchy wastes. Bioresour. Technol..

[149] Thornton R.J. (1985). The introduction of flocculation into a homothallic wine yeast. A practical example of the modification of winemaking properties by the use of genetic techniques. Am. J. Enol. Vitic..

[150] Fogel S., Welch J.W., Cathala G., Karin M. (1983). Gene amplification in yeast: CUP1 copy number regulates copper resistance. Curr. Genet..

[151] Kutyna D.R., Varela C., Stanley G.A., Borneman A.R., Henschke P.A., Chambers P.J. (2012). Adaptive evolution of *Saccharomyces cerevisiae* to generate strains with enhanced glycerol production. Appl. Microbiol. Biotechnol..

[152] Moneke A., Okolo B., Nweke A., Ezeogu L., Ire F. (2010). Selection and characterisation of high ethanol tolerant Saccharomyces yeasts from orchard soil. Afr. J. Biotechnol..

[153] Jones R.M., Russell I., Stewart G.G. (1986). The use of catabolite derepression as a means of improving the fermentation rate of brewing yeast strains. J. Am. Soc. Brew. Chem..

[154] Fukuda H., Kizaki Y., Tsukihashi T., Wakabayashi S. (2001). Shochu brewing characteristics and properties of a trichothecin-resistant shochu yeast mutant. Biotechnol. Lett..

[155] Mukai N., Nishimori C., Fujishige I.W., Mizuno A., Takahashi T., Sato K. (2001). Beer brewing using a fusant between a sake yeast and a brewer’s yeast. J. Biosci. Bioeng..

[156] Zhang J., Zhang C., Qi Y., Dai L., Ma H., Guo X., Xiao D. (2014). Acetate ester production by Chinese yellow rice wine yeast overexpressing the alcohol acetyltransferase-encoding gene ATF2. Genet. Mol. Res..

[157] Guangfa X., Zhiqiang Z., Jin M.A., Lan W., Jianwei F.U. (2010). Screening of yeast strains forquick fermentation in Chinese rice wine brewing. China Brew..

[158] Yang L., Jiang Y., Li Y. (2013). Screening, identification and fermentation characteristics of a Chinese rice wine yeast strain with high stress tolerance. J. Chin. Inst. Food Sci. Technol..

[159] Hirooka K., Yamamoto Y., Tsutsui N., Tanaka T. (2005). Improved production of isoamyl acetate by a sake yeast mutant resistant to an isoprenoid analog and its dependence on alcohol acetyltransferase activity, but not on isoamyl alcohol production. J. Biosci. Bioeng..

[160] Inoue T., Iefuji H., Katsumata H. (2012). Characterization and isolation of mutants producing increased amounts of isoamyl acetate derived from hygromycin B-resistant sake yeast. Biosci. Biotechnol. Biochem..

[161] Takahashi T., Ohara Y., Sueno K. (2017). Breeding of a sake yeast mutant with enhanced ethyl caproate productivity in sake brewing using rice milled at a high polishing ratio. J. Biosci. Bioeng..

[162] Pires E.J., Teixeira J.A., Brányik T., Vicente A.A. (2014). Yeast: The soul of beer’s aroma—A review of flavour-active esters and higher alcohols produced by the brewing yeast. Appl. Microbiol. Biotechnol..

[163] Krogerus K., Magalhães F., Vidgren V., Gibson B. (2015). New lager yeast strains generated by interspecific hybridization. J. Ind. Microbiol. Biotechnol..

[164] Xu Y., Zhao G.A., Wang L.P. (2006). Controlled formation of volatile components in cider making using a combination of *Saccharomyces cerevisiae* and *Hanseniaspora valbyensis* yeast species. J. Ind. Microbiol. Biotechnol..

[165] Valles B.S., Bedriñana R.P., Tascón N.F., Simón A.Q., Madrera R.R. (2007). Yeast species associated with the spontaneous fermentation of cider. Food Microbiol..

[166] Bellon J.R., Eglinton J.M., Siebert T.E., Pollnitz A.P., Rose L., de Barros Lopes M., Chambers P.J. (2011). Newly generated interspecific wine yeast hybrids introduce flavour and aroma diversity to wines. Appl. Microbiol. Biotechnol..

[167] Bellon J.R., Yang F., Day M.P., Inglis D.L., Chambers P.J. (2015). Designing and creating Saccharomyces interspecific hybrids for improved, industry relevant, phenotypes. Appl. Microbiol. Biotechnol..

[168] Peng Q., Meng K., Zheng H., Yu H., Zhang Y., Yang X., Lin Z., Xie G. (2022). Metabolites comparison in post-fermentation stage of manual (mechanized) Chinese Huangjiu (yellow rice wine) based on GC-MS metabolomics. Food Chem. X.

[169] Yang Y., Xia Y., Wang G., Tao L., Yu J., Ai L. (2019). Effects of boiling, ultra-high temperature and high hydrostatic pressure on free amino acids, flavor characteristics and sensory profiles in Chinese rice wine. Food Chem..

